# Distribution of Cytokines and Their Receptors Targeted by Biologics in the Treatment of Psoriasis

**DOI:** 10.1177/12034754251408380

**Published:** 2026-01-26

**Authors:** Nardin Hanna, Sabrina B. Yang, Meghan L. McPhie, Mark G. Kirchhof

**Affiliations:** 1 Faculty of Medicine, University of Ottawa, Ottawa ON, Canada; 2 Division of Dermatology, Department of Medicine, University of Ottawa and the Ottawa Hospital, Ottawa ON, Canada

**Keywords:** psoriasis, biologics, cytokines, RNA, protein, distribution

## Abstract

Biologic therapies have revolutionized the management of psoriasis. These agents are often assumed to share similar risk profiles despite targeting distinct cytokines with diverse mechanisms of action. The aim of this study was to evaluate the distribution of cytokines and their receptors targeted by current biologic therapies in order to identify tissues potentially subject to immunomodulation and to better understand potential targets of the biologics. Using publicly available RNA and protein expression databases, we analyzed expression patterns of TNF-α, IL-12, IL-23, IL-17, and their corresponding receptors. Expression profiles varied considerably in both RNA levels and tissue distribution. Some correlations were observed between cytokine expression sites and the adverse event profiles of biologics. Further research is required to validate these associations and to clarify the tissue-specific effects of cytokine-targeted therapies in psoriasis.

## Introduction

Since the development of the first humanized monoclonal antibody in 1996, biologics have become a fundamental element of the treatment of many autoimmune conditions including rheumatoid arthritis, ulcerative colitis/Crohn’s disease, and systemic lupus erythematous.^1^ In 2002, biologics designed to treat psoriasis spearheaded the introduction of these protein-based therapeutics for the treatment of immune-mediated skin diseases.^2^ There are now 11 FDA-approved biological drugs for the treatment of psoriasis, with a significant number of new biologics under consideration.^3^ Each of these biological therapeutics (all in the form of monoclonal antibodies) targets 1 or more of the following pro-inflammatory cytokines implicated in manifesting psoriatic skin changes: TNF-α, IL-12, IL-23, and IL-17.^3^

The introduction of these medications has revolutionized the treatment of psoriasis, changing therapeutic targets from 50% to 90% improvement with respect to the baseline Psoriasis Area and Severity Index score.^4,5^ A small number of these systemic immunomodulators have been associated with potential adverse outcomes including opportunistic infections (TB, fungal infections), malignancies (lymphoma, nonmelanoma skin cancer), other immune-mediated conditions (multiple sclerosis and lupus), and heart disease (congestive heart failure).^6^ All biologics have been assumed to carry the similar safety risk profile and are used with the same precautions, despite the diverse mechanisms of action of the cytokines targeted.

The purpose of this article was to review the distribution of the cytokines and their corresponding receptors targeted by biological therapies within human tissues. Our goal was to elucidate which tissues may be targeted by biologics. This information may be used to identify potentially immunomodulated tissues with the use of biological therapies. Using expression information, and subsequent clinical trial evaluation, it may be possible to use this information to correlate the side effect profile of biologics. This information may also later be applied to stratify biological treatments on the basis of phenotype/genotype of psoriasis or medical history/comorbidity profile of patients.

We conducted a search in a basic RNA and protein mapping database (The Human Protein Atlas (HPA), version 19.3; and Ensembl, version 92.38) for genes coding cytokines TNF-α, IL-12, IL-23, IL-17, and their receptors.^7^ These genes were as follows: TNF, TNFRSF1A (TNFR1), TNFRSF1B (TNFR2), IL-12A, IL-12B, IL-12RB1, IL-12RB2, IL-23A, IL-23R, IL-17A, IL-17F, IL-17RA, and IL-17RC. Consensus transcript expression levels were used, sourced from 3 tissue gene datasets using normalized expression values: HPA, Genotype-Tissue Expression, and Functional Annotation of Mammalian Genomes 5 (FANTOM5).^7^ When considering group tissue expression, the individual tissue site with the highest normalized transcript expression was used to quantify group RNA/protein production.

Tissue groups were divided as follows in RNA: brain (olfactory bulb, cerebral cortex, hippocampal formation, amygdala, basal ganglia, hypothalamus, thalamus, midbrain, pons and medulla, spinal cord, and cerebellum); eye (retina); muscle (smooth, skeletal, cardiac); endocrine tissues (adrenal gland, parathyroid gland, thyroid gland, pituitary gland); lung; gastrointestinal tract (tongue, esophagus, salivary gland, stomach, duodenum, small intestine, colon, rectum); lymphoid tissues and bone marrow (bone marrow, thymus, tonsils, lymph nodes, spleen, appendix); pancreas; liver and gallbladder; kidney; male tissues (testis, epididymis, prostate, seminal vesicle, ductus deferens); female tissues (breast, vagina, uterine cervix, endometrium, fallopian tube, ovary, placenta); skin; adipose and soft tissue; blood cells (B cells, T cells, NK cells, monocytes, granulocytes, dendritic cells, and total blood mononuclear cells).^7^

RNA tissue expression was classified as any of the following, as per the HPA subcategories: tissue enhanced (at least 4 times higher mRNA level in 1 tissue site than the average level in all other tissues); group enriched (at least 4 times higher average mRNA level in a group of 2 to 5 tissues than any other tissue); tissue enriched (at least 4 times higher mRNA level in any 1 tissue than any other tissues).^7^

## RNA and Protein Expression of Cytokines

### TNF-α

TNF-α is a pro-inflammatory cytokine encoded by the TNF gene that signals through TNFR1 (TNFRSF1A) and TNFR2 (TNFRSF1B). It plays a central role in psoriasis pathophysiology by activating dermal dendritic cells and Th17 cells, promoting the release of additional pro-inflammatory cytokines. The therapeutic relevance of TNF-α in psoriasis was first identified through the clinical success of TNF inhibitors.^8,9^ Currently, 5 TNF-α inhibitors are FDA and Health Canada-approved for psoriasis/psoriatic arthritis: adalimumab, certolizumab, etanercept, golimumab, and infliximab.

RNA expression of TNF was detected in nearly all tissues, with the highest individual tissue expression in the spleen (Supplemental Figure 1A). Tissue-enhanced expression was noted in bone marrow and lymphoid tissues, with other high-expression groups including blood cells, lung, and gastrointestinal tract. Lowest RNA expression was observed in endocrine tissues, male reproductive tissues, and pancreas. Protein data for TNF were unavailable, precluding RNA-protein correlation (Supplemental Figure 1B).

TNFR1 (TNFRSF1A) exhibited highest RNA expression in granulocytes, though overall tissue specificity was low, with consistent expression across multiple tissue groups. Highest group expression was noted in blood cells, gastrointestinal tract, and liver, while lowest expression occurred in pancreas, skin, and brain. Protein expression largely correlated with RNA, except in soft tissues, where protein was detected despite low RNA, and in blood cells, where protein data were missing.

TNFR2 (TNFRSF1B) showed maximal RNA expression in monocytes, with tissue-enhanced expression in blood cells. Other high-expression tissues included bone marrow, lymphoid tissues, adipose tissue, and female reproductive tissues. Low expression was observed in pancreas, endocrine tissues, and skin. Protein expression data were limited and poorly correlated, with no detectable protein in annotated tissues.

When considered together, RNA expression of TNF, TNFR1, and TNFR2 was highest in lymphoid tissues, bone marrow, blood cells, gastrointestinal tract, adipose tissue, liver, lung, and female reproductive tissues. Protein expression data, though limited, generally mirrored RNA trends, with exceptions in liver, ovary, and vagina.

High TNF and receptor expression in these tissues likely reflects sites where TNF-α is critical to T cell–mediated immune responses. Clinically, this is consistent with the known side effect profile of TNF inhibitors, including respiratory infections (bacterial/viral pneumonia, tuberculosis reactivation), hepatitis B/C reactivation, disseminated infections, septicemia, and bacteremia, corresponding to high expression in lungs, liver, and blood cells, respectively.^10-14^ There is also a potential increased risk of lymphoproliferative malignancies, aligning with tissue-enhanced expression in bone marrow and blood cells.^12^ Nonmelanoma skin cancers have been noted to be increased in psoriasis patients compared with rheumatoid arthritis patients despite low TNF expression in healthy skin suggesting potentially other contributing factors to this observation.^15-18^ Table 1 summarizes the top RNA-expressing tissues for each cytokine/receptor, the corresponding biologics, and their reported side effect profiles.

**Table 1.** Top Tissues With Highest RNA Expression of Cytokines/Receptors Targeted by Biologics in Psoriasis, the Corresponding Biologics, and Key Reported Side Effects.

Cytokine/receptor	Top tissues (RNA)	Biologic	Side effect profile
TNF-α	Spleen, appendix, tonsil, bone marrow, monocytes, lungs, lymph nodes, T cells, tongue, small intestine	Certolizumab, adalimumab, etanercept, golimumab, infliximab	Pneumonia, TB reactivation, hepatitis B/C reactivation, sepsis, lymphoproliferative cancers, nonmelanoma skin cancer^10-14^
IL-12/IL-23	Thymus, T cells, tonsils, testis, B cells, tongue, lymph node, urinary bladder, esophagus, PBMCs	Ustekinumab	Upper respiratory tract infections, nasopharyngitis, headache, arthralgia^38^
IL-23	T cells, tonsil, thymus, testis, B cells, tongue, lymph node, bladder, esophagus, PBMCs	Guselkumab, risankizumab, tildrakizumab	Infections, inflammation, allergic reactions, nausea, vomiting, liver problems, and immune reactions^39^
IL-17A	Tongue, tonsil, urinary bladder, appendix, esophagus, small intestine, gallbladder, lung, T cells, vagina	Secukinumab, ixekizumab	*Candida* infections, URIs, IBD exacerbation, neutropenia^27-30^
IL-17A/IL-17F	Tongue, tonsil, bladder, appendix, esophagus, small intestine, lung, T cells, cervix, gallbladder	Bimekizumab	Increased fungal infections (thrush, intertrigo, vulvovaginal candidiasis), URIs, COVID-19, pyelonephritis, cellulitis, folliculitis^34^
IL-17RA	Thymus, bone marrow, lymph node, breast, spleen, adipose tissue, lung, granulocytes, appendix	Brodalumab	Infections, nasopharyngitis, headache, neutropenia, IBD exacerbation, arthralgia, potential suicide risk^35-37^

RNA expression data were obtained from the Human Protein Atlas, GTEx, and FANTOM5.

Abbreviations: FANTOM5, Functional Annotation of Mammalian Genomes 5; GTEx, Genotype-Tissue Expression; IBD, inflammatory bowel disease; PBMCs, peripheral blood mononuclear cells; URI, upper respiratory infection.

These findings suggest that certain tissues may be disproportionately immunosuppressed by TNF inhibitors due to tissue-specific TNF expression. However, in some high-expression tissues, such as the gastrointestinal tract and female reproductive organs, evidence for increased infection or malignancy is limited, highlighting that high RNA or protein expression does not necessarily translate to higher clinical risk. Noninfectious adverse effects—including nausea, abdominal pain, headache, congestive heart failure, pancytopenia, and aplastic anemia—also correspond to tissues with significant TNF expression.^10^ Understanding these expression profiles may assist clinicians in tailoring biologic therapy to individual comorbidity profiles or family histories.

### IL-12 and IL-23

IL-12 and IL-23 are heterodimeric cytokines that share a common subunit encoded by IL-12B, and thus, their expression must be considered together. IL-12 is composed of subunits encoded by IL-12A and IL-12B, while IL-23 is composed of subunits encoded by IL-23A and IL-12B. Their receptors are also heterodimers: IL-12 signals through IL-12RB1 and IL-12RB2, whereas IL-23 signals through IL-12RB1 and IL-23R. Both cytokines are implicated in psoriasis pathogenesis through regulation of Th1 and Th17 cell differentiation, activation of keratinocytes, and amplification of proinflammatory signalling.^9^ Current biologics either target IL-23 exclusively or both IL-12 and IL-23 together; no biologics selectively target IL-12 alone.^12^ Four agents are FDA/Health Canada-approved: guselkumab, risankizumab, tildrakizumab, and ustekinumab.

RNA expression of IL-12A was highest in the esophagus, with group-enhanced expression in the gastrointestinal tract, particularly proximal digestive tissues (Supplemental Figure 2A). Additional expression was noted in brain tissues, female reproductive tissues (fallopian tube), lung, and blood cells, whereas expression was lowest in skin, eye, and liver/gallbladder. No protein expression of IL-12A was detected (Supplemental Figure 2B).

RNA expression of IL-12B was highest in the thymus, with tissue-enhanced expression in lymphoid tissues. Other high-expression groups included blood cells, endocrine tissues, and skin. Protein expression was strongest in endocrine, bone marrow, and lymphoid tissues, with additional signal in brain, gastrointestinal tract, male and female tissues.

RNA expression of IL-23A was highest in T cells, with group-enhanced expression in blood cells, lymphoid tissues, and male tissues (testis; Supplemental Figure 3A). Other notable expression occurred in the proximal gastrointestinal (GI) tract (tongue, esophagus), kidney, and bladder. Protein expression was partially correlated, with significant levels in bone marrow, lymphoid tissues, and testis, though data in blood cells were lacking (Supplemental Figure 3B).

The shared receptor subunit IL-12RB1 showed highest RNA expression in T cells, with tissue-enhanced expression in blood cells and lymphoid tissues. Protein expression was not reported.

IL-12RB2 RNA expression was highest in NK-cells, with tissue-enhanced expression in blood cells and pancreas. Additional expression occurred in placenta, skeletal and cardiac muscle, and brain tissues. Protein expression (based on antibody staining) correlated with RNA, with high levels in pancreas and brain, and moderate levels in female tissues and muscle.

IL-23R RNA was highest in T cells, with tissue-enhanced expression in blood cells and lymphoid tissues, and additional signal in endocrine tissues, male tissues, and GI tract. Protein expression was weaker, with low but detectable expression in bone marrow/lymphoid tissues, endocrine, male, and GI tissues.

Cumulative RNA expression of IL-12A, IL-12B, IL-12RB1, and IL-12RB2 was highest in blood cells, bone marrow/lymphoid tissues, pancreas, GI tract, brain, female tissues, and lung. Protein expression largely paralleled this, with strongest signals in brain, female reproductive tissues, endocrine tissues, lymphoid tissues, and GI tract.

For IL-23A, IL-12B, IL-12RB1, and IL-23R, cumulative RNA expression was highest in blood cells, bone marrow/lymphoid tissues, male tissues, GI tract, kidney/bladder, lung, and endocrine tissues, with partial correlation to protein expression.

Unlike TNF inhibitors, there is no robust evidence that ustekinumab (IL-12/IL-23 blockade) increases the risk of infection or malignancy in patients with psoriasis, despite theoretical concerns.^19-21^ This may reflect the relatively lower normalized RNA expression values for IL-12/IL-23 than for TNF. For example, the highest expression for a TNF receptor (TNFRSF1A) reached 86.5 normalized units, whereas the highest for IL-12/IL-23 was 28.6 (IL-12RB1), with most transcripts between 0 and 10.22. This lower baseline expression may translate to less clinical immunosuppression.

The IL-23 inhibitors (guselkumab, risankizumab, tildrakizumab) were approved in 2017 to 2018 and therefore have the least long-term safety data. Current evidence suggests no significant increase in serious infections, tuberculosis reactivation, opportunistic infections, malignancies, IBD, or suicidal ideation.^22,23^ It is plausible that their favorable safety profile is partly due to the low normalized RNA expression of IL-23 and its receptors, resulting in less off-target immunosuppression compared with TNF blockade.

### IL-17A and IL-17F

The IL-17 family of cytokines are central mediators in psoriasis, with IL-17A and IL-17F being the most relevant subtypes.^24^ Both signal through the IL-17 receptor complex (IL-17RA/IL-17RC), which is expressed on a wide range of epithelial and stromal tissues. IL-17A is the most biologically potent, inducing downstream gene activation several fold higher than IL-17F. At mucosal and epithelial surfaces, IL-17A drives chemokine production, recruiting and activating neutrophils, which can further amplify IL-17 signalling. In psoriasis, IL-17A and IL-17F promote keratinocyte hyperproliferation and abnormal differentiation, downregulate barrier proteins such as filaggrin, and enhance the local proinflammatory cascade.^25^

Currently, 2 IL-17A inhibitors are approved for the treatment of psoriasis: ixekizumab and secukinumab.^8^ In addition, brodalumab, an IL-17 receptor A antagonist that blocks signalling of IL-17A, IL-17F, and the IL-17A/IL-17F heterodimer, is also approved.^8^ Most recently, bimekizumab, the first monoclonal IgG antibody to target both IL-17A and IL-17F simultaneously, received FDA and Health Canada approval for the treatment of moderate-to-severe plaque psoriasis.^26^

RNA expression of IL-17A was highest in the tongue, with group-enriched expression in the gastrointestinal tract (predominantly tongue), urinary bladder, and lymphoid tissues (Supplemental Figure 4A). Expression was minor to absent in most other tissue groups. Protein expression did not correlate with RNA data, as no detectable protein was reported in any tissues (Supplemental Figure 4B).

For IL-17F, RNA expression was most prominent in the tonsils, followed by the bladder, esophagus, and appendix, with negligible expression in other organs. No protein expression was detected in the tissues examined.

The highest RNA expression of IL-17RA was observed in the thymus, with tissue-enhanced expression in lymphoid tissues. Additional high expression was noted in female reproductive tissues, adipose/soft tissue, and lung. Low expression was found in the eye, urinary bladder, kidney, and pancreas. Protein expression was only partially correlated: High protein levels were reported in the thyroid gland, pancreas, bone marrow, and lymphoid tissues, despite low or variable RNA expression in some of these sites.

RNA expression of IL-17RC was broadly distributed across tissues without tissue specificity, with the highest levels detected in the liver. Tissue groups with the highest expression included gastrointestinal tract, liver and gallbladder, endocrine tissues, and male reproductive tissues. Lowest expression was noted in red blood cells, bone marrow, lymphoid tissues, and lung. Protein expression was partly correlated, with high protein levels in endocrine and gastrointestinal tissues, but conflicting data in bone marrow and lymphoid tissues (low RNA but high protein), and in the liver (high RNA but low protein).

Cumulative expression of IL-17A, IL-17F, IL-17RA, and IL-17RC was highest in bone marrow and lymphoid tissues, liver and gallbladder, female reproductive tissues, and the gastrointestinal tract. Protein expression only partially correlated with RNA data, with the strongest protein expression observed in lymphoid tissues, endocrine tissues, the gastrointestinal tract, and the pancreas.

The 2 IL-17A antagonists, ixekizumab and secukinumab have not been associated with an increased risk of serious infection or cardiovascular events. Risk assessment for tuberculosis reactivation has also been negative. However, these agents are associated with a modest increase in nonserious infections, particularly upper respiratory tract infections (eg, nasopharyngitis, rhinorrhea, influenza, pharyngitis, oropharyngitis, pharyngolaryngeal pain) as well as Candida infections.^27-29^ Other reported adverse events include exacerbation of inflammatory bowel disease and neutropenia.^30^ These findings may correspond to the relatively high RNA expression of IL-17 in the lungs, gastrointestinal tract, and skin. The neutropenia observed may be explained by IL-17’s role in neutrophil development and trafficking.^30^ Although IL-17 expression is also enriched in the liver, an increased risk of hepatitis reactivation has not been demonstrated.^21,31,32^ High RNA expression of IL-17 and its receptors in breast tissue could theoretically predispose to infection or malignancy; however, current evidence suggests that IL-17 may play a pro-oncogenic and pro-metastatic role in breast cancer such that inhibition may reduce rather than increase risk.^33^ Ultimately, larger and longer term studies are needed to clarify the infection and malignancy risks associated with IL-17 inhibitors.

Bimekizumab, the first biologic to simultaneously target IL-17A and IL-17F, has been associated with an increased risk of fungal infection, reported in up to 27% of patients.^34^ The most frequent presentations included oral thrush, intertrigo, and vulvovaginal candidiasis, with some patients developing multiple concurrent infections. Additional reported infections included upper respiratory tract infections, COVID-19, and other bacterial infections. These adverse events may be explained by the tissue distribution of IL-17A and IL-17F, which show the highest RNA expression in the tonsils, bladder, esophagus, appendix, and tongue sites that overlap with the mucosal and urinary tract infections observed clinically.

Although brodalumab also targets the IL-17 pathway, it must be considered separately as it antagonizes the IL-17RA receptor, which binds multiple ligands (IL-17A, IL-17C, IL-17E, and IL-17F).^35^ Brodalumab has been associated with serious infections, including fungal infections such as cryptococcal meningitis, along with increased rates of nasopharyngitis, neutropenia, headache, arthralgia, and exacerbation of inflammatory bowel disease.^35-37^ Concerns have also been raised regarding an elevated risk of suicide, although the supporting evidence remains limited.^35^ The observed neutropenia may be attributable to IL-17RA antagonism, given IL-17’s role in neutrophil maturation and trafficking.^30^ The risks of headache, meningitis, and suicidality may relate to the high expression of IL-17RA in the central nervous system, while the increased risk of inflammatory bowel disease flares corresponds with the strong protein expression of IL-17RA within gastrointestinal tissues. By contrast, the higher frequency of nasopharyngitis is less clearly explained by expression data, as protein levels of IL-17RA were undetectable in the nasopharynx. Collectively, these findings highlight how the broader blockade of IL-17 signalling by brodalumab may contribute to a distinct side effect profile compared with IL-17A-specific inhibitors.

## Conclusion

In this article, we reviewed available RNA and protein expression data of cytokines targeted by biologics in the context of psoriatic disease. The expression profiles of the cytokines targeted in psoriasis biologic therapies—TNF-α, IL-12, IL-23, and IL-17—were diverse in both RNA levels and tissue distribution. We identified potential correlations between cytokine expression patterns and the side effect profiles of biologic therapies. Further high-powered and long-term studies are needed to confirm these associations. Our review has several limitations: Protein expression data are difficult to standardize across cytokines, and some protein data were missing or inconclusive, so comparisons were primarily based on RNA expression. Future studies should update these findings as more comprehensive protein data become available. Clinically, these findings may help predict tissue-specific risks of biologic therapies, guide monitoring strategies, and inform personalized treatment decisions for patients with psoriasis.

## Supplemental Material

sj-docx-1-cms-10.1177_12034754251408380 – Supplemental material for Distribution of Cytokines and Their Receptors Targeted by Biologics in the Treatment of Psoriasis Supplemental material, sj-docx-1-cms-10.1177_12034754251408380 for Distribution of Cytokines and Their Receptors Targeted by Biologics in the Treatment of Psoriasis by Nardin Hanna, Sabrina B. Yang, Meghan L. McPhie and Mark G. Kirchhof in Journal of Cutaneous Medicine and Surgery
