# Supplementary material for: Distribution of Cytokines and Their Receptors Targeted by Biologics in the Treatment of Psoriasis
Source: J Cutan Med Surg. 2026 Jan 26;30(4):409–15. doi: 10.1177/12034754251408380 (PMC13478408; doi:10.1177/12034754251408380)
Supplement: Supplementary file 1 [file sj-docx-1-cms-10.1177_12034754251408380.docx]

**SUPPLEMENTARY TABLE 1**

**Total number of palliative sedation procedures reported in Quebec during the study period ^31, 39-43^**

| **Year** | **Number of patients who received Palliative Sedation** |
| --- | --- |
| 2015-7 | 1080 |
| 2017-8 | 745 |
| 2018-9 | 1243 |
| 2019-20* | 1631 |
| 2020-1 | 1539 |
| 2021-2 | 1838 |
| **Total** | 8076 |

* (in the 2020-1 report)
